# How Reliable Are ATP Bioluminescence Meters in Assessing Decontamination of Environmental Surfaces in Healthcare Settings?

**DOI:** 10.1371/journal.pone.0099951

**Published:** 2014-06-18

**Authors:** Navid Omidbakhsh, Faraz Ahmadpour, Nicole Kenny

**Affiliations:** 1 Research & Development, Virox Technologies, Inc., Oakville, Ontario, Canada; 2 Professional & Technical Services, Virox Technologies Inc., Oakville, Ontario, Canada; University Paris South, France

## Abstract

**Background:**

Meters based on adenosine triphosphate (ATP) bioluminescence measurements in relative light units (RLU) are often used to rapidly assess the level of cleanliness of environmental surfaces in healthcare and other settings. Can such ATP measurements be adversely affected by factors such as soil and cleaner-disinfectant chemistry?

**Objective:**

This study tested a number of leading ATP meters for their sensitivity, linearity of the measurements, correlation of the readings to the actual microbial contamination, and the potential disinfectant chemicals’ interference in their readings.

**Methods:**

First, solutions of pure ATP in various concentrations were used to construct a standard curve and determine linearity and sensitivity. Serial dilutions of a broth culture of Staphylococcus *aureus*, as a representative nosocomial pathogen, were then used to determine if a given meter’s ATP readings correlated with the actual CFUs. Next, various types of disinfectant chemistries were tested for their potential to interfere with the standard ATP readings.

**Results:**

All four ATP meters tested herein demonstrated acceptable linearity and repeatability in their readings. However, there were significant differences in their sensitivity to detect the levels of viable microorganisms on experimentally contaminated surfaces. Further, most disinfectant chemistries tested here quenched the ATP readings variably in different ATP meters evaluated.

**Conclusions:**

Apart from their limited sensitivity in detecting low levels of microbial contamination, the ATP meters tested were also prone to interference by different disinfectant chemistries.

## Introduction

Several types of pathogens can readily survive on high-touch environmental surfaces in healthcare and other settings [1]–[4] as a result these surfaces may act as vehicles for the spread of a variety of nosocomial pathogens [3], [5]. In 2002 in the US, 5% of all patients acquired such infections and of these, the mortality rate was nearly 6% [6]–[11]. In the United States alone, the cost of such hospital-acquired infections (HAIs) is estimated to be between 5 and 29 billion dollars annually [9], [12]–[14].

To limit the impact of HAIs, routine cleaning and disinfection of high-touch environmental surfaces in healthcare facilities is crucial for infection control [2], [4], [15]–[19]. In addition, it is imperative to ascertain that the decontamination procedures in such facilities are optimal. While the widely used practice of visual inspections may be sufficient for aesthetic purposes, it does not provide quantitative feedback on the effectiveness of the decontamination process [7], [20], [21]. While culture-based approaches provide quantitative results, they cannot provide immediate feedback and antibody- or PCR-based techniques have limited applications such as in the food industry where immediate availability of the results may be less crucial [2], [13].

ATP bioluminescence meters, which measure the concentration of ATP as relative light units (RTU) in organic material and living cells [16], are widely used in food and beverage industries because of their ease of use and fast turn-around of results. Such meters are increasingly being used in healthcare facilities as well.

This study evaluated four leading ATP bioluminescence monitoring systems for their accuracy and linearity in detecting ATP values, detection limits for microbial count, correlation with plate-counting using *Staphylococcus aureus* and the quenching and enhancement effect of various disinfectant chemistries.

## Materials and Methods

### Test Materials

#### ATP bioluminescence meters.

Kikkoman Lumitester PD-20 from Luminultra Technologies Ltd. (with LuciPac Pen swabs), EnSURE Hygiene Meter – ATP-205 from Hygiena/Scigiene Corporation (with ATP3000 SuperSnap swabs), Clean-Trace NG Luminometer UNG2 from 3M Company (with Surface ATP - UXL100 swabs), and Charm novaLUM from Charm Sciences Inc. (with PocketSwab Plus ATP swabs).

#### ATP standard solution

Adenosine 5′-triphosphate, disodium salt (ATP.2Na) from Enzo Life Sciences.

#### Microorganism

Staphylococcus aureus (ATCC 6538).

#### Culture medium

4% Tryptone soya agar (TSA) plates (Oxoid Microbiology Products; Nepean, Ontario).

### Disinfectants Tested

Table 1 shows the list of tested disinfectants in this study. They were selected because they are sold for the decontamination of environmental surfaces in healthcare settings. In addition to the commercial products, a few antimicrobial active ingredients were also used in this study to compare their results with actual disinfectant formulations.

**Table 1:** Tested disinfectants, their active ingredients, and manufacturers.

Product	Chemical Ingredients as listed on the Label	Manufacturer, Location
CaviCide	Isopropyl alcohol, 17.2%; 2-butoxyethanol,1–5%; Diisobutyl –phenoxy-ethoxy-ethyl-dimethyl-benzyl ammonium chloride, 0.28%	Metrex; Orange, CA
CleanCide	Citric acid, 0.6%	Wexford Labs, Inc.;Kirkwood, MO
Ultra Clorox Bleach (1∶10 dilution)	Sodium hypochlorite, 5–8%	The Clorox Company;Oakland, CA
PCS 1000	Sodium hypochlorite, 0.1%	Process Cleaning Solutions Ltd.;Peterborough, ON
Sani-Cloth Plus	Isopropanol, 10–20%; 2-butoxyethanol, 1–4%; Benzyl-C12–18-alkyldimethylammonium chlorides <0.125%, C12–18-alkyl[(ethylphenyl) methyl] dimethyl chlorides,<0.125%	Nice-Pak ProductsInc.; Mooresville, IN
Clorox Hydrogen Peroxide Wipes	Hydrogen peroxide, 1.4%;	The Clorox Company;Oakland, CA
Clorox Clean-up disinfectant	Sodium hypochlorite, 1.84%	The Clorox Company;Oakland, CA
Isopropyl alcohol	Isopropyl alcohol, 70% v/v	VWR International,LLC.; Mississauga, ON
Hydrogen peroxide	Hydrogen peroxide, 0.5% w/w	Arkema Inc.;Philadelphia, PA
BTC 50 (1∶125 dilution)	Alkyl dimethyl benzyl ammonium chloride(C12–18) 50–51.5%, Ethanol 5–5.5%	Stepan Company; Northfield, IL
Accel TB	Hydrogen peroxide, 0.5%	Virox TechnologiesInc.; Oakville, ON
Accel PREVention RTU	Hydrogen peroxide, 0.5%	Virox TechnologiesInc.; Oakville, ON
Virox 5 RTU	Hydrogen peroxide, 0.5%	Sealed AirCorporation; Elmwood Park, NJ
Sporicidin	Phenol, 1.58%, sodium phenate, 0.06%	Sporicidin by Contec Inc.; Spartanburg, SC

### Methods

First, the ATP luminometer meters were tested for their linearity in reading standard ATP solutions. A 0.1 molar solution of ATP standard powder was prepared in autoclaved deionized (DI) water, followed by serial 10-fold dilutions from 10^−2^ to 10^−10^. 10 µL of each dilution was pipetted directly onto the swab tip using positive displacement tips. This was done to avoid the variability resulting from the difference of swab-to-swab efficiency in picking up the organic load from the surface. Each meter measured ATP and reported the data in RLU. Later, serial dilutions of *S. aureus* were prepared from a freshly thawed stock culture. A 10 µL volume of each serial dilution (10^0^ to 10^8^) was separately pipetted directly on the tip of each swab and the readings were recorded. To correlate the RLU reading with the actual CFU, 900 µL of 10^−9^ and 10^−10^ dilutions of the bacterial suspension were separately plated on TSA in triplicates and incubated for 24 hours at 36±1°C. Any chemical interference through quenching or enhancement of bioluminescence was tested by placing 10 µL of the appropriate dilution of ATP standard solution onto the tip of a swab followed by placement of 10 µL of the test disinfectant. The baseline ATP solution concentrations used above were individually determined for each of the luminometers, selecting the aliquot with ATP concentration that fell between the ATP meters’ true maximum and minimum detection limits based on their obtained linearity standard curves. Also, the volume of dispensed disinfectant on the swabs, 10 µL, was determined by testing the average volume of water required to keep 50% of a 10 cm×10 cm hard non-porous surface (a typical surface area dimension recommended by ATP meter manufacturers to be swabbed) wet for 3 minutes. The calculated average volume required was 80 µL in ambient room temperatures. This volume was reduced to 10 µL to compensate for the evaporation of the volatile ingredients.

To account for the repeatability of the results, all the tests have been performed in triplicates.

### Statistical Analysis

Microsoft Excel was used in this study to determine correlation, R^2^, between mean readings. A log transformation of the RLU and CFU values were used since the original distribution is highly skewed with a long tail towards the higher values. Therefore, geometric mean is used for these calculations.

## Results

**Figure 1:**
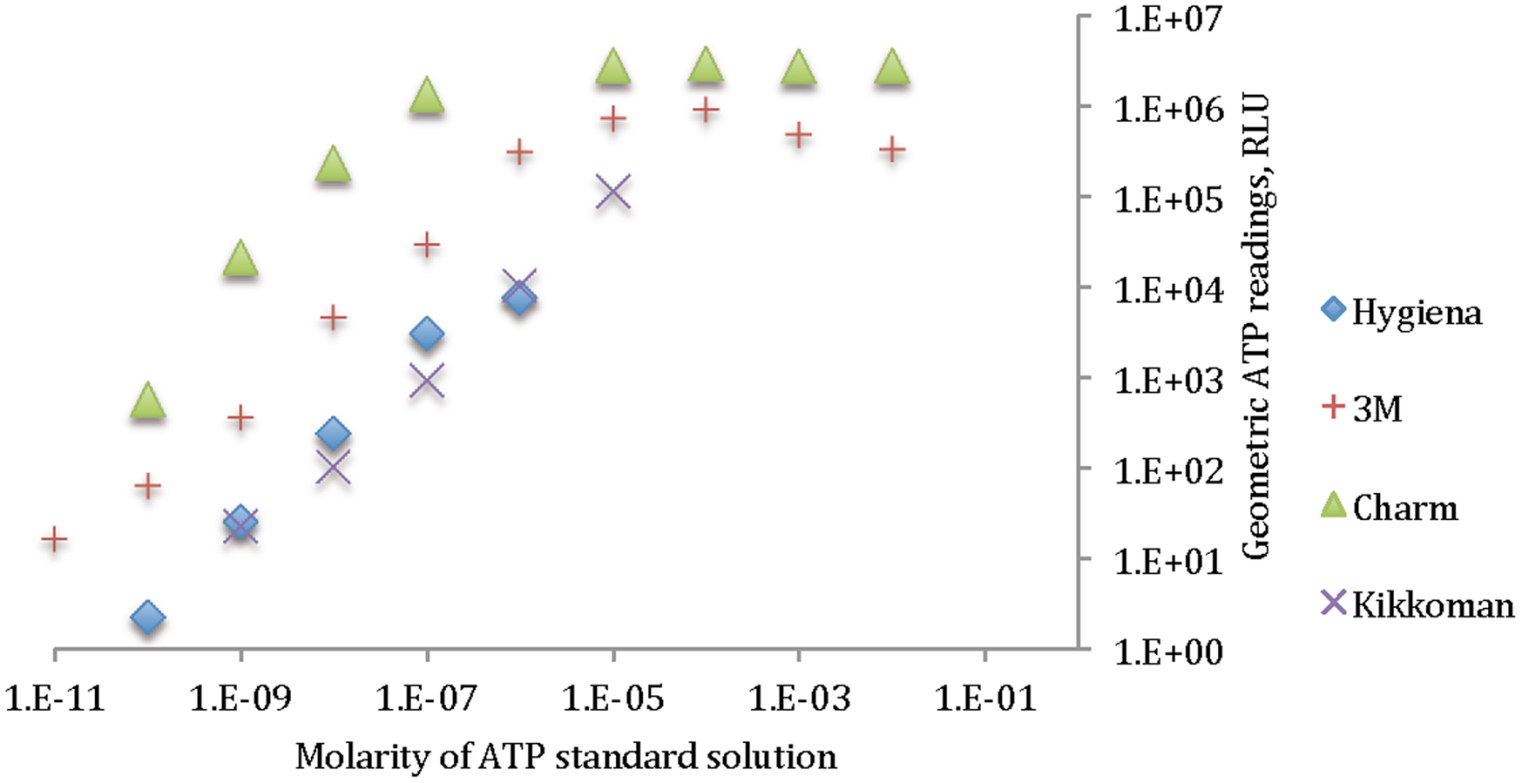
Linearity in ATP readings for 4 different ATP meters.

Figure 1 shows the linearity between the geometric mean of the ATP readings versus the molarity of ATP standard solution.

As can be seen, none of the ATP meters provided a linear relationship between ATP readings and the actual concentration of the ATP throughout the whole test range. Based on the results, approximately 6 logs of ATP reading RLUs is the highest difference observed in reading the same ATP concentration among different ATP meter brands. It can be noticed, however, that at some selected regions, the readings are almost linear; for example, for Hygiena, if the ATP reading at the 10^−6^ molarity is not considered, the rest of the data are completely linear (R^2^ of 0.99952 compared to 0.98591 for the dataset including 10^−6^ molarity data point). Table 2 shows the correlation of ATP values to the ATP readings both at logarithmic scales.

**Table 2:** Correlation between ATP amount and ATP reading values in logarithmic scales for 4 different ATP meters.

	Charm	Hygiena	3M	Kikkoman
Correlation	0.8230	0.9827	0.9228	0.9966

**Figure 2:**
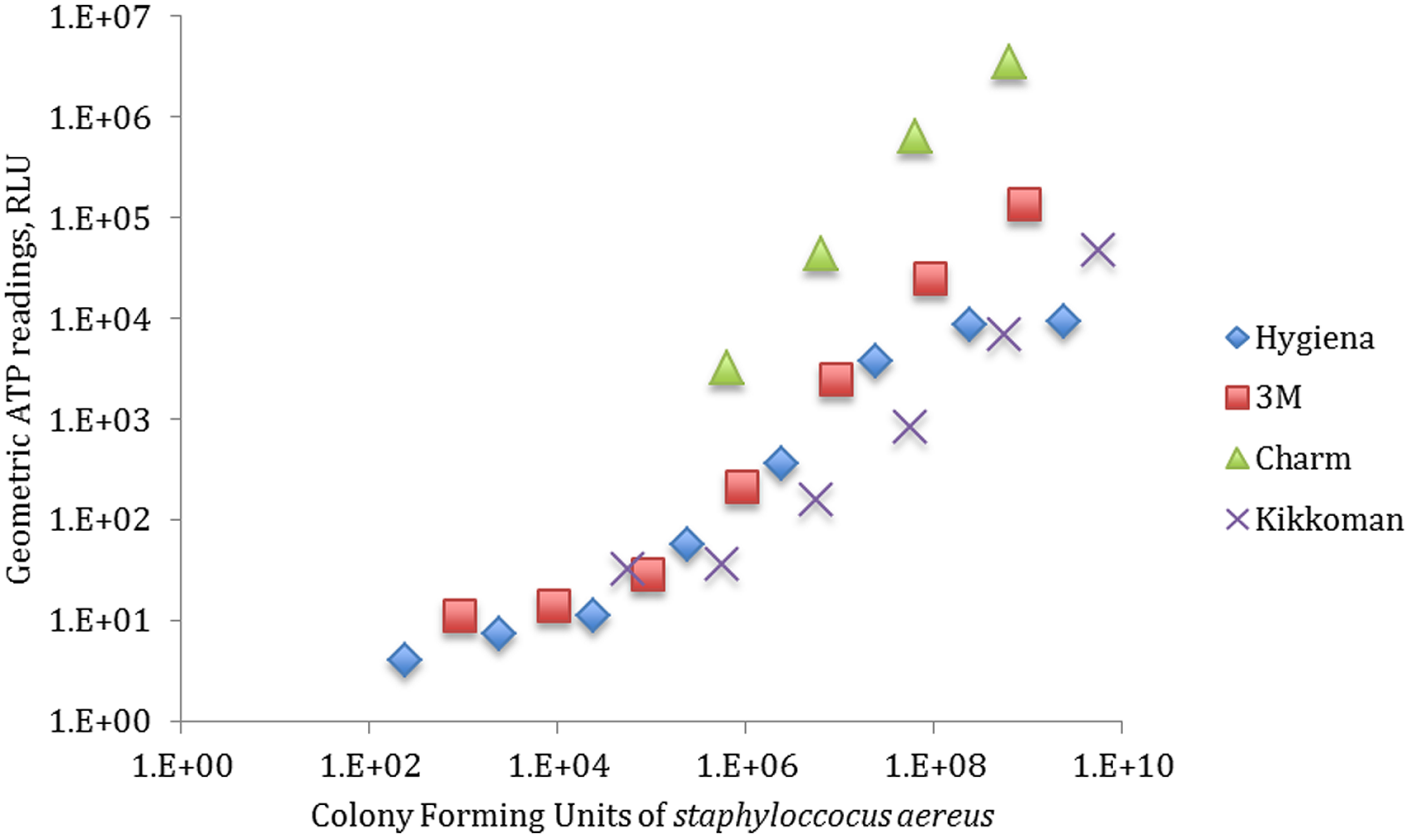
Geometric mean of ATP readings for various dilutions of *S. aureus* CFUs.

Figure 2 shows CFUs of *S. aureus* versus the geometric mean of the ATP readings for each ATP meter.

The detection limit of each ATP meter is displayed in Figure 2, as well as the smallest detectable number of the test organism on the swab. Table 3 shows the exact values of CFU at which each bioluminator was able to detect. It also demonstrates the correlation between RLU reading to CFUs.

**Table 3:** The minimum CFU of *S. aureus* that was detected for each ATP meter.

	Charm	Hygiena	3M	Kikkoman
Least detected CFU count	6.17E+05	2.40E+02	8.98E+02	5.60E+04
Correlation of RLU readings to plate counting (both in logarithmic scales)	0.9955	0.97737	0.9746	0.95634

**Figure 3:**
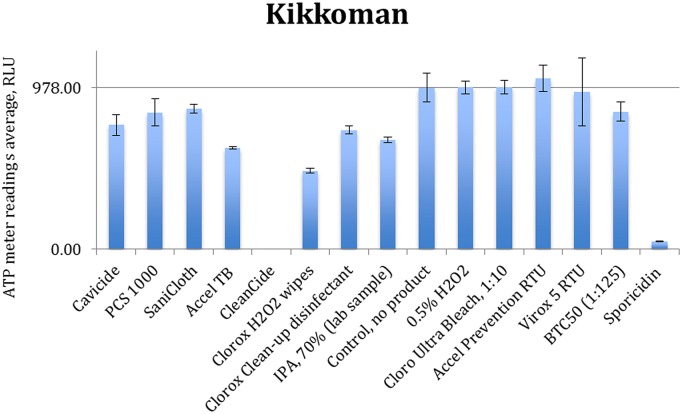
Quenching and enhancement effects of various disinfectant chemistries on Kikkoman luminometer readings, the control was ATP standard solution with 10^−7^ molarity.

**Figure 4:**
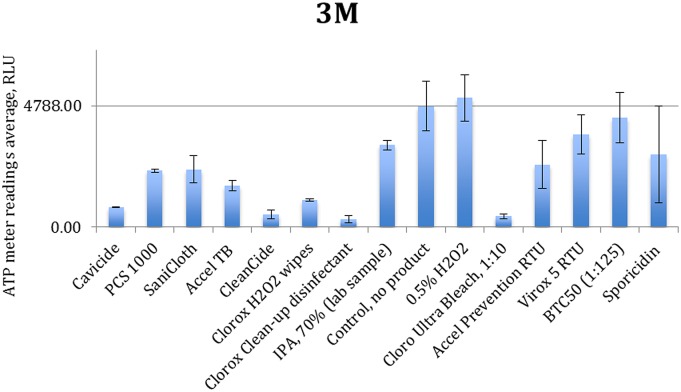
Quenching and enhancement effects of various disinfectant chemistries on 3M luminometer readings. the control was ATP standard solution with 10^−8^ molarity.

**Figure 5:**
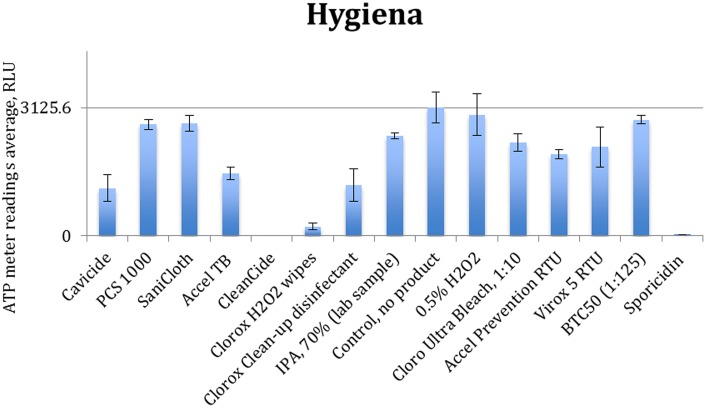
Quenching and enhancement effects of various disinfectant chemistries on Sciegiene luminometer readings, the control was ATP standard solution with 10^−7^ molarity.

**Figure 6:**
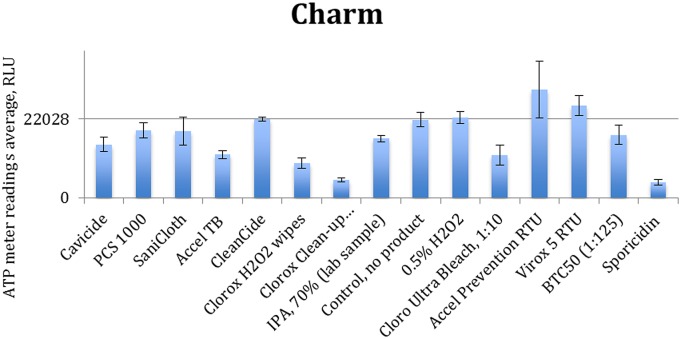
Quenching and enhancement effects of various disinfectant chemistries on Charm luminometer readings, the control was ATP standard solution with 10^−9^ molarity.

Figures 3 to 6 show the quenching/enhancement effect of each disinfectant on the ATP readings.

In Figures 3 to 6, the horizontal line represents the average ATP reading for the control sample, which is a dilution of the standard ATP solution and is specified in the caption of each figure. The error bars show the standard deviation for the three measurements at each point. Instances in which the bars which do not reach the horizontal line (even with their error bar) indicate that the disinfectant has significantly quenched the ATP readings.

## Discussion and Concluding Remarks

In spite of the wide acceptance of ATP measurement technology there are gaps in our knowledge concerning the true reliability of the approach to assessing the cleanliness of environmental surfaces in healthcare and other settings. A correlation between RLU and colony forming units (CFU) has been reported previously [15], [17]–[22]. In some studies, ATP meters have not been examined for their correlation with the actual microbial count, and have only reported RLU values as a measure of surface cleanliness [16], [17], [23]. Other studies suggest a loose correlation between the RLU values and the actual counts [1]. Further studies have also shown the interaction of detergents and disinfectants in RLU readings [6], [8], [10], [11], [16], [17], [23] and some include comparisons of different bioluminescent meters [1], [12], [14]. Carrick *et al* compared four different ATP meters and their swab units and found poor detection and linearity when the surfaces are swabbed. They also reported that the swabbing units are unreliable at picking up total surface ATP. In a study by Carmen and colleagues, two of the three tested ATP luminometer failed verification, which means that they both need modifications by their manufacturers. In this study, four of the market leading ATP meters were used. Disinfectant chemistries used in this study are the most widely used products in North America. They include quaternary ammonium chlorides, phenol, sodium hypochlorite, isopropanol, citric acid and hydrogen peroxide. Furthermore, individual active ingredients of these disinfectants were tested in parallel to see the interference of the whole formulation versus the active ingredient(s).

Our analyses demonstrate that the higher the concentration of ATP or *S. aureus* on the swab tip, the higher the ATP reading values; in other words there is a strong positive correlation between true concentrations and RLU readings. These results support earlier studies showing such a correlation [6], [8], [10], [11], [15], [17]–[19] [12], [14], [20], [21]. The observed correlations were slightly higher for the standard ATP solutions than those derived from *S. aureus*. This slight lower RLU value correlation can be explained by the fact that a single bacterial cell of a specific strain does not always produce/release the same amount of ATP molecules at a given time.

The detection limit test also showed that there could be a significant difference in the level of bacteria detectable by each device. For example, one device required 6.17×10^5^ CFU on the swab in order to detect an RLU value of greater than zero. Minimum detection limit values among different brands varies at up to 2 logs of ATP standard dilution. The same for maximum ATP concentration true detection varies at up to 2 logs as well. Therefore, in actual testing, an ATP reading of zero by swabbing may be misleading since the surface may in fact contain at least 10^2^ CFU bacteria. It should be noted that the detection limit results in this study are based on *S. aureus*, while in real life, many other bacteria may be present in the environment and therefore the lower limit of bacterial detection varies very more widely.

Comparing Figures 1 and 2, we see that the detection limits of each luminometer for the bacterial ATP and the standard ATP solution are completely different. For example, Figure 1 shows that 3M detects ATP at 10^−11^ molarity, Charm and Hygiena detect it at 10^−10^ molarity and Kikkoman at 10^−9^ molarity, therefore 3M can detect the least concentration of ATP among these four bioluminescence meters, while by examining Figure 2, it can be seen that Hygiena is the most sensitive unit among the four in detecting *S. aureus* by showing a lowest detection limit of 2.4×10^2^ CFU followed by 3M (8.98×10^2^ CFU), Kikkoman (5.6×10^4^CFU) and Charm (6.2×10^5^ CFU). The only conformance between these two sets of data is the detection limit for Charm, which is the last in both cases.

Chemical disinfectants seem to significantly affect the ATP readings of all four tested units. Data in Figures 3 to 6 are summarized in Table 4.

**Table 4:** Quenching and enhancing summary for tested disinfecting chemistries versus each tested ATP unit.

Product	Kikkoman	3M	Hygiene	Charm
CaviCide	23.18%	83.67%	62.80%	31.61%
PCS 1000	15.34%	53.49%	12.94%	13.71%
Sani-Cloth Plus	13.16%	52.23%	12.05%	14.78%
Accel TB	37.08%	65.69%	51.44%	44.48%
CleanCide	99.86%	89.64%	99.90%	-0.43%
Clorox Hydrogen Peroxide Wipes	51.40%	77.95%	92.83%	55.34%
Clorox Clean-up disinfectant	26.28%	93.54%	60.40%	77.02%
IPA, 70%	32.34%	32.20%	22.07%	24.24%
0.5% H2O2	−0.10%	−6.79%	5.43%	−2.80%
Ultra Clorox Bleach (1∶10)	−0.20%	91.18%	27.35%	45.34%
Accel PREVention RTU	−5.69%	48.29%	36.11%	−38.80%
Virox 5 RTU	2.69%	23.34%	30.73%	−17.88%
BTC 50 (1∶125)	14.79%	9.54%	9.54%	19.34%
Sporicidin	95.16%	40.14%	99.10%	80.15%

Enhancing is shown in negative values while quenching is in positive values.

These data were generated based on the deviation of the average ATP readings from the control sample. The values in this table represent the percentage deviation from the control sample. Since the majority of the tests led to quenching, the quenching values are shown in positive while enhancements are demonstrated as negative.

These results clearly show that each chemistry has a unique effect in either quenching or enhancing the ATP readings. Some formulations (Sporicidin and CleanCide) show the highest quenching among all tested chemicals. The 3M-meter is also shown to be the most susceptible to disinfectant chemistries. Comparing 0.5% hydrogen peroxide (in DI water) with disinfectant products containing hydrogen peroxide (0.5–1.4%), we observe that other ingredients (inerts) present in these formulations are almost entirely responsible for the interaction. Comparing Accel TB, Accel PREVention RTU and Virox 5 RTU shows that although they all have 0.5% hydrogen peroxide as actives, their different inert ingredients can have a profound distinctive effect in the interference.

CleanCide (0.6% Citric acid) and Sporicidin (phenol based) have the most quenching effect among all the products. The CleanCide data are in conformance with findings of Mubiru [15], [17]–[19], [24] and that citric acid interferes with ATP determination by bioluminescence. Phenol was not tested in this study separately and therefore it is not possible to conclude whether Sporidicin interference comes from phenol or the inert ingredients in the formulation. Cavicide and Sani-Cloth plus are both combinations of quaternary ammonium compounds, 2-butoxy ethanol, and isopropanol, with close concentration ranges. These show almost identical quenching results with Cavicide to have more quenching effect on Hygiene and Charm units. This could be due to using different types quaternary ammonium compounds and/or using different types of inert chemicals. BTC 50, another Quat based disinfectant was also tested here to examine the effect of an exemplary quaternary ammonium compound. It shows mild quenching on Kikkoman and Charm and no effect on Hygiene and 3M. If it is used in a disinfectant formulation however, it may show different interaction, due to the effect of the other ingredients in its formulation.

By examining Table 1, it can be seen that healthcare disinfectants contain high levels of active ingredients. They also have other inert ingredients, which are usually not disclosed on their materials safety data sheets. Therefore swabbing a surface which has already been treated with a disinfectant has the potential to introduce high levels of residual chemicals to the swab and, subsequently, to the ATP measuring device. In food processing facilities, on the other hand, the chemical exposure will be significantly lower as FDA requirements (21 CFR 178.1005 & 1010 and similar guidelines) significantly limit the level of chemicals in food sanitizing and disinfecting solutions. This, results in much less chemical interaction, which could be the reason why not much chemical interaction is reported in ATP bioluminescence meters in these applications. It should be noted however that in this study, the disinfectant was directly applied to swab for the interaction test, while in real life situations, the disinfectant will be applied to the surface first, and in most part it will dry before swabbing. Therefore for volatile active ingredients such as alcohols or hydrogen peroxide, the actual chemical interaction may be less than the test results here, but for those non-volatile active ingredients, such as quaternary ammonium compounds or citric acid, the chemical interaction should be more or less the same if the surface is properly swabbed.

In summary, these results suggest that ATP meters cannot be relied upon to evaluate the effective disinfection of a healthcare surface and in particular, cannot be used as a tool to compare the effectiveness of disinfection between different disinfectants. These units have a number of limitations in detecting the true number of organisms on the surface, which can lead into false confidence in surface disinfection. Furthermore the cleaning/disinfecting chemistry residues can have a very high impact in the ATP readings, and therefore again can result in more false confidence. As of now, there have been no reports of scientific publications that specifically studied the quenching phenomena for its true cause. Our assumption on the mechanism of chemical quenching points to two main directions: either the chemicals react with the ATP molecules and make them no longer available by breaking/masking the ATP molecule, or perhaps the chemicals enter the luciferase activity chamber and adversely affect the enzymatic pathway for fluorescence generation. In this study, all the test solutions such as ATP standard solutions, inoculum and disinfectant chemistries were pipetted into the swab, and therefore the efficiency of each swab was not studied here. Furthermore, only one type of Gram-positive bacterium was tested here to obtain more definitive and reliable conclusions. Further studies should involve the use of both Gram-positives and Gram-negatives to expand on this study’s finding. Needless to say, testing viral contaminations with ATP meters would result futile as viral cells do not contain or produce ATP molecules on their own, raising another concern on the limitations of the ATP bioluminescence technology in healthcare use.

Our findings suggest that introducing ATP meters to healthcare facilities, as a disinfection validation tool is not a reliable choice. The limitations of ATP luminometers clearly show that the units are not reliable in confirming proper removal of disease causing agents at healthcare settings. Inaccurate bioluminescence results causing false confidence on surface disinfection can ultimately jeopardize public health and rise infection control costs at hospitals and healthcare facilities.

We should note that our findings are based on one bacterium namely *S.aureus*, on four ATP meters and fourteen disinfectant products. All tests were performed in controlled laboratory conditions. To avoid variability in the recovery, the inoculum was directly applied to the swabs, and as such our tests did not involve sampling of environmental surfaces.

Our findings, in conjunction with the available literature, can help healthcare infection control practitioners make more educated decisions about the methods they choose to evaluate the microbial cleanliness of healthcare surfaces.

## References

[1] Havill NL, Havill HL, Mangione E, Dumigan DG, Boyce JM (2011) Cleanliness of portable medical equipment disinfected by nursing staff. American Journal of Infection Control 39: 602–604 10.1016/j.ajic.2010.10.030 21496956

[2] Dancer SJ (2009) The role of environmental cleaning in the control of hospital-acquired infection. Journal of Hospital Infection 73: 378–385 10.1016/j.jhin.2009.03.030 19726106

[3] Kramer A, Schwebke I, Kampf G (2006) How long do nosocomial pathogens persist on inanimate surfaces? A systematic review. BMC Infect Dis 6: 130 10.1186/1471-2334-6-130 16914034 PMC1564025

[4] Rampling A, Wiseman S, Davis L, Hyett AP, Walbridge AN, et al (2001) Evidence that hospital hygiene is important in the control of methicillin-resistant Staphylococcus aureus. Journal of Hospital Infection 49: 109–116 10.1053/jhin.2001.1013 11567555

[5] Dancer S (1999) Mopping up hospital infection. Journal of Hospital Infection 43: 85–100. 10549308 10.1053/jhin.1999.0616

[6] Green T, Russell S (1998) Effect of chemical sanitizing agents on ATP bioluminescence measurements. Journal of Food. 10.4315/0362-028x-61.8.1013 9713763

[7] Griffith CJ, Cooper RA, Gilmore J, Davies C, Lewis M (2000) An evaluation of hospital cleaning regimes and standards. Journal of Hospital Infection 45: 19–28 10.1053/jhin.1999.0717 10833340

[8] Green TA, Russell SM, Fletcher DL (1999) Effect of chemical cleaning agents and commercial sanitizers on ATP bioluminescence measurements. Journal of Food Protection 174 62: 86–90. 10.4315/0362-028x-62.1.86 9921836

[9] Hassan M, Tuckman HP, Patrick RH, Kountz DS, Kohn JL (2010) Cost of Hospital-Acquired Infection. Hospital Topics 88: 82–89 10.1080/00185868.2010.507124 20805070

[10] Velazquez M (1997) Quenching and enhancement effects of ATP extractants, cleansers, and sanitizers on the detection of the ATP bioluminescence signal. j food prot 60: 799–803. 10.4315/0362-028X-60.7.799 31026891

[11] Brown E, Eder AR, Thompson KM (2010) Do surface and cleaning chemistries interfere with ATP measurement systems for monitoring patient room hygiene? The Journal of hospital infection 74: 193–195. 20060618 10.1016/j.jhin.2009.10.006

[12] Carrick K, Barney M, Navarro A (2001) The comparison of four bioluminometers and their swab kits for instant hygiene monitoring and detection of microorganisms in the brewery. Journal of the Institute of Brewing 107: 31–37.

[13] Velusamy V, Arshak K, Korostynska O, Oliwa K, Adley C (2010) An overview of foodborne pathogen detection: In the perspective of biosensors. Biotechnology Advances 28: 232–254 10.1016/j.biotechadv.2009.12.004 20006978

[14] Sciortino CV, Giles A (2012) Validation and comparison of three adenosine triphosphate luminometers for monitoring hospital surface sanitization: A Rosetta Stone for adenosine triphosphate testing. American Journal of Infection Control 40: e233–e239 10.1016/j.ajic.2012.04.318 23021416

[15] Turner D, Daugherity E, Altier C (2010) Efficacy and Limitations of an ATP-Based Monitoring System. Journal of the American. PMC2846007 20353694

[16] Bellamy E (2012) An audit of cleaning effectiveness using adenosine triphosphate (ATP) bioluminescence assay following outbreaks of infection. Journal of Infection Prevention 13: 154–157 10.1177/1757177412455835

[17] Anderson RE, Young V, Stewart M, Robertson C, Dancer SJ (2011) Cleanliness audit of clinical surfaces and equipment: who cleans what? Journal of Hospital Infection 78: 178–181 10.1016/j.jhin.2011.01.030 21497943

[18] Mulvey D, Redding P, Robertson C, Woodall C, Kingsmore P, et al (2011) Finding a benchmark for monitoring hospital cleanliness. Journal of Hospital Infection 77: 25–30 10.1016/j.jhin.2010.08.006 21129820

[19] Lewis T, Griffith C, Gallo M, Weinbren M (2008) A modified ATP benchmark for evaluating the cleaning of some hospital environmental surfaces. Journal of Hospital Infection 69: 156–163 10.1016/j.jhin.2008.03.013 18468725

[20] Murphy SC, Kozlowski SM, Bandler DK, Boor KJ (1998) Evaluation of Adenosine Triphosphate-Bioluminescence Hygiene Monitoring for Trouble-Shooting Fluid Milk Shelf-Life Problems. Journal of Dairy Science 81: 817–820 10.3168/jds.S0022-0302(98)75639-5 9565886

[21] Chen F-C, Godwin SL (2006) Comparison of a rapid ATP bioluminescence assay and standard plate count methods for assessing microbial contamination of consumers’ refrigerators. j food prot 69: 2534–2538. 17066941 10.4315/0362-028x-69.10.2534

[22] Leon MB, Albrecht JA (2007) Comparison of adenosine triphosphate (ATP) bioluminescence and aerobic plate counts (APC) on plastic cutting boards*. Journal of Foodservice 18: 145–152.

[23] Moore G, Smyth D (2012) The Use of ATP Bioluminescence to Assess the Efficacy of Modified Cleaning Programs: A Potential Problem Encountered within the Intensive Care Setting. AJIC: American Journal of Infection Control 37: 1–2. 10.1016/j.ajic.2010.02.011 20605265

[24] Mubiru DN, Coyne MS, Grove JH (2008) Citric Acid Interferes with Adenosine Triphosphate Determination by Bioluminescence. Analytical Letters 41: 2587–2594 10.1080/00032710802363339

